# Halofuginone dually regulates autophagic flux through nutrient-sensing pathways in colorectal cancer

**DOI:** 10.1038/cddis.2017.203

**Published:** 2017-05-11

**Authors:** Guo-Qing Chen, Rui-Hong Gong, Da-Jian Yang, Ge Zhang, Ai-Ping Lu, Siu-Cheong Yan, Shu-Hai Lin, Zhao-Xiang Bian

**Affiliations:** 1 Laboratory of Brain and Gut Research, Center for Clinical Research on Chinese Medicine, School of Chinese Medicine, Hong Kong Baptist University, Hong Kong SAR, China; 2 Chongqing Academy of Chinese Materia Medica, Chongqing, China; 3 Department of Applied Biology and Chemical Technology, Hong Kong Polytechnic University, Hung Hom, Kowloon, Hong Kong; 4 Department of Biochemistry and Molecular Cell Biology, Shanghai Key Laboratory for Tumor Microenvironment and Inflammation, Shanghai Jiao Tong University School of Medicine (SJTU-SM), Shanghai 200025, China

## Abstract

Autophagy has a key role in metabolism and impacts on tumorigenesis. Our previous study found that halofuginone (HF) exerts anticancer activity in colorectal cancer (CRC) by downregulating Akt/mTORC1 (mechanistic target of rapamycin complex 1) signaling pathway. But whether and how HF regulates autophagy and metabolism to inhibit cancer growth remains an open question. Here, we unveil that HF activates ULK1 by downregulation of its phosphorylation site at Ser757 through Akt/mTORC1 signaling pathway, resulting in induction of autophagic flux under nutrient-rich condition. On the other hand, HF inactivates ULK1 by downregulation of its phosphorylation sites at Ser317 and Ser777 through LKB1/AMPK signaling pathway, resulting in autophagic inhibition under nutrient-poor condition. Furthermore, Atg7-dependent autophagosome formation is also induced under nutrient-rich condition or blocked in nutrient-poor environment, respectively, upon HF treatment. More interestingly, we also found that HF inhibits glycolysis under nutrient-rich condition, whereas inhibits gluconeogenesis under nutrient-poor condition in an Atg7-dependent manner, suggesting that autophagy has a pivotal role of glucose metabolism upon HF treatment. Subsequent studies showed that HF treatment retarded tumor growth in xenograft mice fed with either standard chow diet or caloric restriction through dual regulation of autophagy *in vivo*. Together, HF has a dual role in autophagic modulation depending on nutritional conditions for anti-CRC.

Macroautophagy (referred to hereafter as autophagy) is a catabolic cellular recycling process, which is a routine ‘garbage disposal’ service to cells, eliminating damaged components that could otherwise become toxic. It involves the sequestration of cytoplasmic material within double-membraned organelles, so-called autophagosomes, followed by lysosomal digestion.^1, 2^ To monitor autophagy, the measurement of the conversion of the soluble form of LC3 (named LC3-I) to the phosphatidylethanolamine-bound LC3 (LC3-II) by immunoblot is a reliable indicator of autophagic activity. However, this method could not estimate overall autophagic flux or rate of flow, as LC3-II is both induced and degraded during autophagy. Therefore, it would be necessary to conduct several approaches to analyze autophagy in mammalian cells, including immunoblotting analysis of LC3 and SQSTM1 (p62), and monitoring autophagosome maturation by tandem mRFP-GFP fluorescence microscopy for autophagic vacuoles.^3, 4^

The role of autophagy in cancer is complex and is likely dependent on tumor type, stage, and genetic context. The current consensus is that autophagy acts as a double-edged sword in cancer. On one hand, autophagy impedes tumor initiation in some models; on the other hand, it can also promote tumorigenesis by facilitating the survival of cancer cells under duress.^5, 6^ The metabolic states also impact the autophagic process. In normal (nutrient-replete) conditions, mechanistic target of rapamycin complex 1 (mTORC1) possesses kinase activity and interacts with a complex that contains ULK1, Atg13, FIP200, and Atg101, which inhibits the initiation of autophagy.^7^ Under metabolic stress, AMP-activated protein kinase (AMPK) acts as a central node that could impinge directly on core components including ULK1 of the autophagy machinery to initiate autophagy.^8^ Notably, AMPK is sensing to nutrient deprivation condition, particularly glucose limitation. On the other hand, the autophagic pathway could also provide metabolic substrate in solid cancers.^9, 10^ In this context, the interplay between autophagy and metabolism is still largely unknown. Nevertheless, many tumors become addicted to autophagy for survival, suggesting inhibition of autophagy as a potential broadly applicable cancer therapy.^11^ The compound SBI-0206965, a highly selective ULK1 kinase inhibitor, regulates autophagy for potentiating cancer treatment.^12^ Another more advanced approach to inhibit autophagy for cancer therapy is the use of hydroxychloroquine to block autophagic flux and cargo degradation and suppress tumor growth,^13^ suggesting that autophagy is a key target for cancer treatment.^14^

Because autophagy serves a dichotomous role in cancer, it would be intriguing to develop an approach to dually regulate autophagy for cancer treatment. In our previous work, the compound halofuginone (HF) was shown to potentiate its bioactivity to inhibit Akt/mTORC1 and retard tumor growth under nutrient-rich condition.^15^ Notably, inhibition of mTORC1 may induce autophagy by phosphorylating ULK1. Thus, we suspected that HF could regulate autophagy in colorectal cancer (CRC) cells. To this end, autophagic flux was investigated in CRC cells upon HF treatment.

Here, we report that HF regulates mTORC1-mediated phosphorylation of ULK1 at Ser757 to initiate autophagy under nutrient-replete condition;^16^ whereas HF inhibits AMPK-mediated phosphorylation of ULK1 at Ser317 and Ser777 to inhibit autophagy under nutrient-poor condition.^17^ Furthermore, we also interrogated the interplay between autophagy and glucose metabolism including glycolysis in nutrient-rich condition as well as gluconeogenesis in nutrient-poor condition, and found that HF modulates glucose metabolism in an ATG7-dependent manner. Moreover, our result also showed that HF dually modulates autophagy to retard tumor growth *in vivo*.

## Results

### HF regulates autophagy through Akt-mTORC1 or LKB1-AMPK signaling pathway

**Figure 1:**
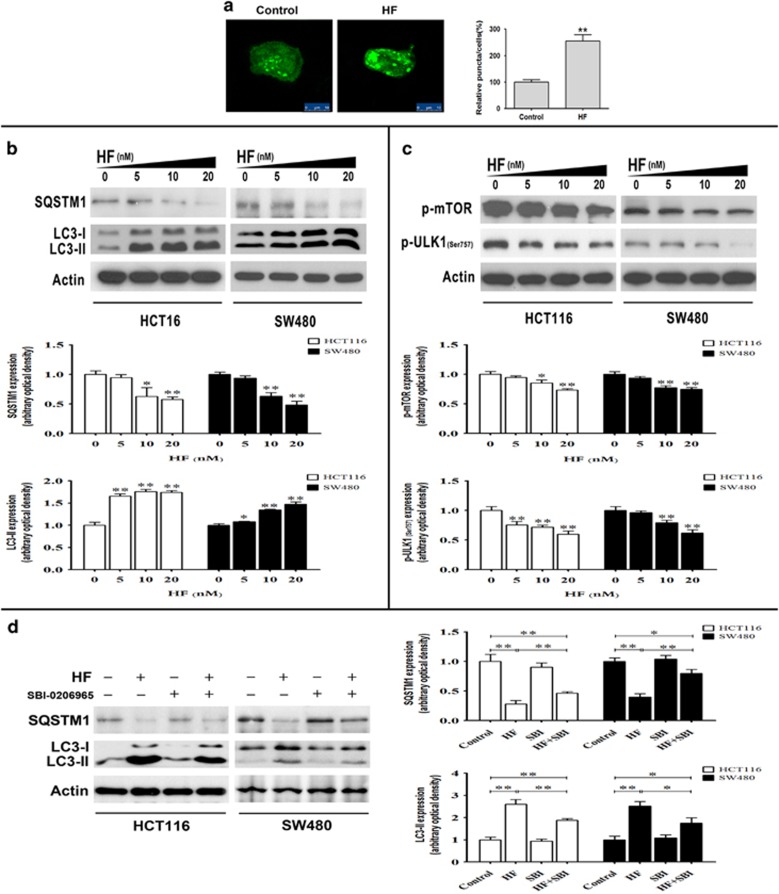
HF induces autophagy in CRC cells under nutrient-rich condition. (**a**) Accumulation of GFP-LC3-II puncta in HCT116 cells with 20 nM HF for 12 h in high-glucose medium. The distribution of GFP-LC3-II was examined by confocal microscope (left panel) and quantitative analysis (right panel). Scale bar: 10 *μ*m. *P<0.05, **P<0.01. (**b**) Protein expressions of SQSTM1 and LC3-II in HCT116 and SW480 cells (upper panel); quantitative analysis of protein levels (bottom panel) treated with 0, 5, 10, 20 nM HF for 12 h in high-glucose medium. *P<0.05, **P<0.01. (**c**) Protein expressions of phospho-mTOR and phospho-ULK1 at Ser757 in HCT116 and SW480 cells (upper panel); quantitative analysis of protein expressions (bottom panel) treated with 0, 5, 10, 20 nM HF for 12 h in high-glucose medium. *P<0.05, **P<0.01. (**d**) Protein expressions of SQSTM1 and LC3-II in HCT116 and SW480 cells (left panel); quantitative analysis of protein expressions (right panel) treated with 20 nM HF or/and 10 *μ*M SBI-0206965 for 12 h in high-glucose medium. *P<0.05, **P<0.01

**Figure 2:**
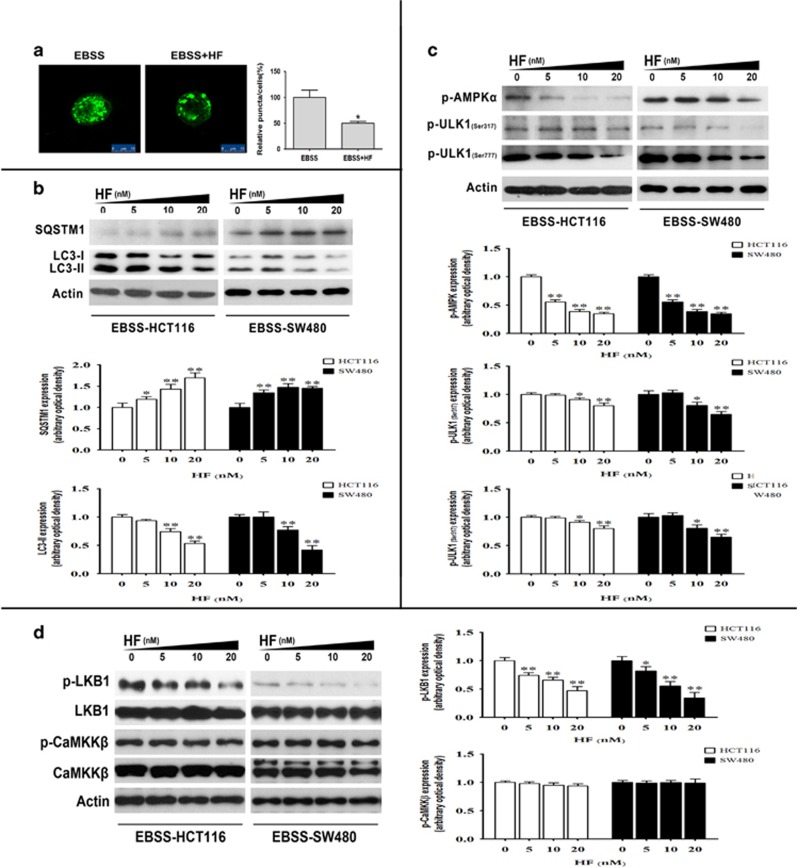
HF inhibits autophagy in CRC cells under nutrient-poor condition. (**a**) Accumulation of GFP-LC3-II puncta in HCT116 cells with 20 nM HF for 2 h in EBSS medium. The distribution of GFP-LC3-II was examined by confocal microscope (left panel) and quantitative analysis (right panel). Scale bar: 10 *μ*m. *P<0.05, **P<0.01. (**b**) Protein expressions of SQSTM1 and LC3-II in HCT116 and SW480 cells (upper panel); quantitative analysis of protein expressions (bottom panel) treated with 0, 5, 10, 20 nM HF for 2 h in EBSS medium. *P<0.05, **P<0.01. (**c**) Protein expressions of phospho-AMPK*α*, phospho-ULK1 at Ser317 and Ser777 in HCT116 and SW480 cells (upper panel); quantitative analysis of protein expressions (bottom panel) treated with 0, 5, 10, 20 nM HF for 2 h in EBSS medium. *P<0.05, **P<0.01. (**d**) Protein expressions of phospho-LKB1, phospho-CaMKK*β* in HCT116 and SW480 cells (left panel); quantitative analysis of protein expressions (right panel) treated with 0, 5, 10, 20 nM HF for 2 h in EBSS medium. *P<0.05, **P<0.01

HF was documented to regulate Akt-mTORC1 signaling in CRC, and mTORC1 also inhibits autophagy through phosphorylation and inactivation of the initiating kinase ULK1 in cancer cells,^18^ prompting us to ask if and how HF regulates autophagy in CRC cells. Thus, GFP-LC3-II, a fluorescent autophagosomal marker, was conducted to monitor autophagy in HCT116 cells. As shown in Figure 1a, we found that HF induced GFP-LC3-II puncta accumulation detected by a confocal microscope in high-glucose medium. To further clarify HF-mediated autophagy, SQSTM1 (p62), which is a putative substrate for evaluating impairment of autophagic degradation, was also determined by western blot assay.^19^ We found that HF significantly reduced SQSTM1, whereas markedly elevated LC3-II protein levels under nutrient-rich condition (Figure 1b). These observations led us to hypothesize that HF induces autophagy in nutrient-rich condition. We further asked how HF initiated autophagy. The western blot assay shown in Figure 1c suggested that HF inhibits mTORC1 and activates ULK1 by downregulating phosphorylation site at Ser757. To validate HF-induced autophagy through ULK1, the highly selective inhibitor SBI-0206965 was used to reduce HF-induced autophagy, indicating that HF regulates autophagy, at least partially, through ULK1 signaling (Figure 1d).^12^ Notably, not only the nutrient-sensing mTORC1, but also AMPK regulates phosphorylation of ULK1 at different sites for autophagic modulation.^17, 18^ In this regard, we further investigated AMPK-mediated phosphorylation at Ser317/Ser777 of ULK1 upon HF treatment under nutrient-poor condition. As we expected, HF declined number of GFP-LC3-II puncta when HCT116 cells were cultured in Earle’s Balanced Salt Solution (EBSS) medium (Figure 2a). Changes in protein levels of SQSTM1 and LC3-II suggested that HF inhibits autophagy under nutrient-poor condition (Figure 2b). As AMPK can directly regulate phosphorylation at Ser317/Ser777 of ULK1, we detected phosphorylated AMPK and phosphorylated ULK1 at Ser317/Ser777, and found that HF downregulated the AMPK-ULK1 signaling pathway in CRC cells cultured in EBSS medium (Figure 2c). The phosphorylation level of acetyl-CoA carboxylase *α* (ACC*α*), a downstream target of AMPK, was also validated (Supplementary Figure S1). To further uncover the mechanism how HF regulates AMPK for autophagy inhibition, we found that LKB1 rather than CaMKK*β* as the upstream event of AMPK, was downregulated when treated with HF (Figure 2d). These results suggested that HF could dually regulate initiation step of autophagy through either Akt-mTORC1-ULK1 or LKB1-AMPK-ULK1 signaling pathways depending on nutritional conditions, by referring to our previous report.^15^

### HF dually modulates autophagosome formation

**Figure 3:**
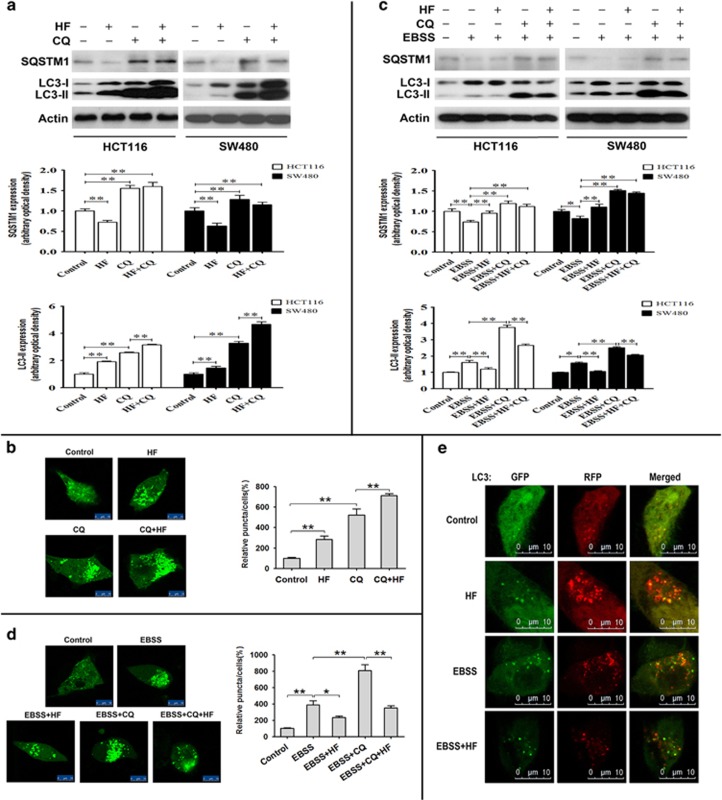
HF dually regulates autophagic flux depending on nutrient conditions. (**a**) Protein expressions of SQSTM1 and LC3-II in HCT116 and SW480 cells (upper panel); quantitative analysis of protein expressions (bottom panel) treated with 20 nM HF or/and 50 *μ*M CQ for 12 h in high-glucose medium. *P<0.05, **P<0.01. (**b**) The abundance of GFP-LC3-II puncta in HCT116 cells with 20 nM HF or/and 50 *μ*M CQ for 12 h in high-glucose medium. The distribution of GFP-LC3-II was examined by confocal microscope (left panel) and quantitative analysis (right panel). Scale bar: 10 *μ*m. *P<0.05, **P<0.01. (**c**) Protein expressions of SQSTM1 and LC3-II in HCT116 and SW480 cells (upper panel); quantitative analysis of protein expressions (bottom panel) treated with 20 nM HF or/and 50 *μ*M CQ for 2 h in EBSS medium. *P<0.05, **P<0.01. (**d**) The abundance of GFP-LC3-II puncta in HCT116 cells with 20 nM HF or/and 50 *μ*M CQ for 2 h in EBSS medium. The distribution of GFP-LC3-II was examined by confocal microscope (left panel) and quantitative analysis (right panel). Scale bar: 10 *μ*m. *P<0.05, **P<0.01. (**e**) HCT116 cells were transfected with mRFP-GFP-LC3 plasmids for 24 h, and then the cells were cultured with 20 nM HF for 12 h in high-glucose medium and 2 h in EBSS medium, separately. The distribution of yellow (autophagosome) and red (autolysosome) puncta was examined by confocal microscope. Scale bar: 10 *μ*m

As mentioned above, HF can dually regulate the initiation step of autophagic flux. We further investigated whether HF could exert a marked impact on later steps including elongation of the double-membrane structure to form the autophagosome under both nutrient-rich and -poor conditions. In this regard, the lysosome inhibitor chloroquine (CQ) that allows the accumulation of the endogenous LC3-II was used to analyze the autophagic flux in CRC cells upon HF treatment. Either HF or CQ caused accumulation of LC3-II puncta and protein expression under nutrient-rich condition (Figures 3a and b). In EBSS medium culture, CQ caused accumulation of LC3-II in CRC cells (Figures 3c and d). Of note, HF greatly synergized with CQ to cause the most accumulation of the marker LC3-II under nutrient-rich condition. Although under nutrient-poor condition, co-treatment of CQ and HF slightly reduced the expression of LC3-II and the decreased the number of GFP-LC3-II puncta. Therefore, we postulated that HF can induce autophagosome formation or inhibit autophagosome membrane elongation, depending on nutrient states.

To further provide evidence of autophagosome formation in autophagic flux, we performed tandem fluorescent-tagged LC3 (mRFP-GFP-LC3) puncta formation assay.^3^ Because GFP loses its fluorescence from deprotonation in acidic lysosomes, autophagosomes display both green and red fluorescence, whereas autolysosomes are red only. Hence, a marked increase in the number of red-only indicates maturation of autophagosomes. As shown in Figure 3e, mRFP-GFP-LC3 aggregated under nutrient-rich condition upon HF treatment, suggesting that HF promotes autophagosome formation. As expected, mRFP-GFP-LC3 also aggregated when nutrient-replete medium was replaced with EBSS medium. However, HF treatment caused both red and yellow fluorescence loss, indicating that HF blocked autophagosome formation at an early stage in EBSS culture.

Because monodansylcadaverine (MDC) is proposed to be an autophagosome indicator, we utilized MDC assay for further investigation of HCT116 cells. As expected, HF induced more green fluorescence accumulation under nutrient-rich condition. By comparing with the CRC cells cultured in EBSS medium without HF treatment, we also observed that EBSS medium induced more green fluorescence, whereas HF reduced green fluorescence (Supplementary Figure S2). Taken together, these results indicate that HF can induce autophagosomal formation when nutrient supply is ample, whereas HF inhibits autophagosomal formation at the early stage of nutrient deprivation.

### HF dually regulates Atg7 for autophagic flux

**Figure 4:**
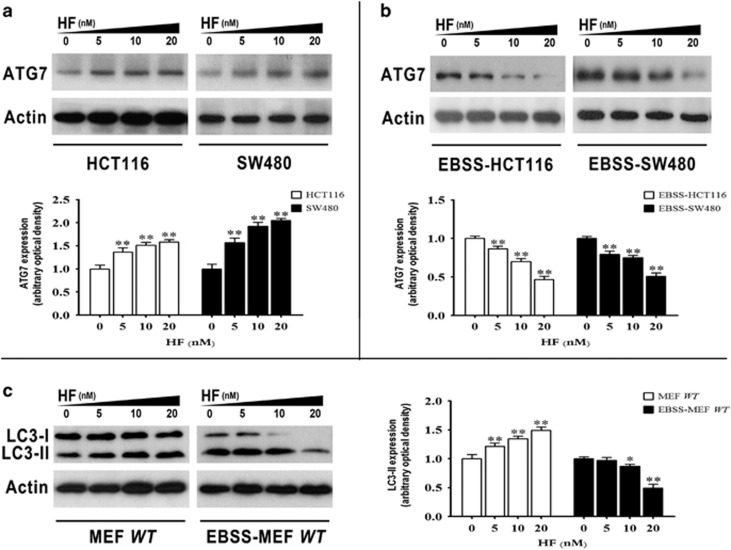
Atg7 is required in HF-modulated autophagy. (**a**) Protein expression of ATG7 in HCT116 and SW480 cells (upper panel); quantitative analysis of protein expressions (bottom panel) treated with 0, 5, 10, 20 nM HF for 12 h in high-glucose medium. *P<0.05, **P<0.01. (**b**) Protein expression of ATG7 in HCT116 and SW480 cells (upper panel); quantitative analysis of protein expressions (bottom panel) treated with 0, 5, 10, 20 nM HF for 2 h in EBSS medium. *P<0.05, **P<0.01. (**c**) Protein expression of LC3-II in WT MEFs (left panel); quantitative analysis of protein expressions (right panel) treated with 0, 5, 10, 20 nM HF for 12 h in high-glucose medium and 2 h in EBSS medium, separately. *P<0.05, **P<0.01

The autophagosomal formation is a key stage in autophagic process and some autophagy-related genes are involved in autophagosome formation, including *Atg5*, *Atg7*, *Atg10,* and *Atg12*. In this regard, we performed quantitative PCR to measure mRNA levels of these autophagy-related genes for a better understanding of molecular events at the stage of autophagosomal formation. Of particular interest, HF treatment pronouncedly enhanced the expression level of *Atg7* under nutrient-rich condition, whereas decreasing the mRNA level of *Atg7* in CRC cells under nutrient-poor condition, but *Atg5*, *Atg10*, and *Atg12* were not significantly altered upon HF treatment (Supplementary Figure S3). Therefore, we further asked whether *Atg7* is required in dual regulation of autophagic flux with HF treatment. By performing western blot analysis, we found that dual regulation of ATG7 in a dose-dependent manner with HF treatment in CRC cells under the two different conditions (Figures 4a and b). Similar to the results of CRC cell treated with HF, the protein level of LC3-II upregulated in WT MEFs under nutrient-rich condition upon HF treatment. Meanwhile, decreased LC3-II expressions were observed in WT MEFs treated with HF under nutrient-poor condition (Figure 4c). The obtained results were consistent with what we had observed in CRC cells treated with HF. However, the exact mechanism of dual regulation of ATG7 in cells depending on nutritional conditions upon HF treatment still needs to be further explored.

### HF regulates glucose metabolism in an Atg7-dependent manner

**Figure 5:**
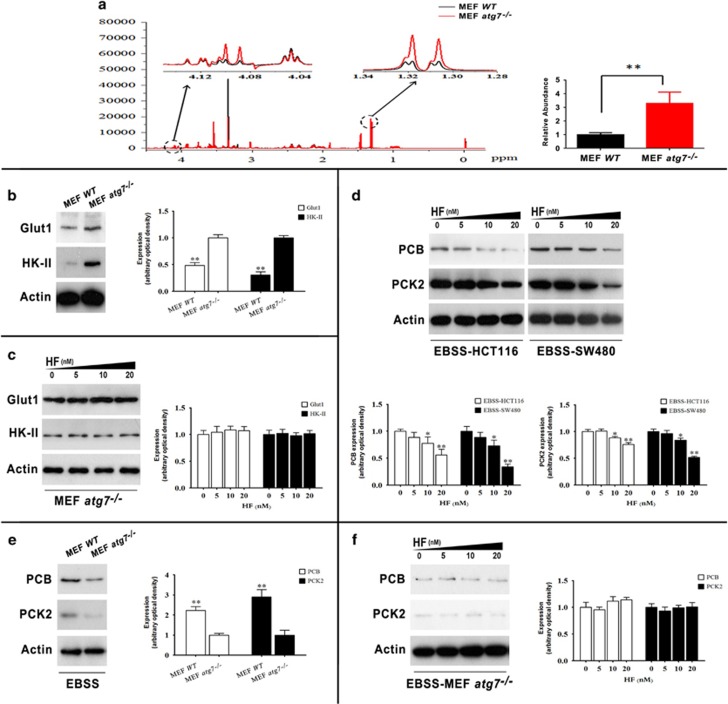
HF regulates glycolysis/gluconeogenesis in an Atg7-dependent manner. (**a**) Lactate signals in WT MEFs and *Atg7*^−/−^ MEFs measured by 1H-NMR spectroscopy (left panel); bar plots of lactate in WT MEFs and *Atg7*^−/−^ MEFs (right panel) (mean±S.E.M., *n*=3). **P<0.01. (**b**) Protein expressions of Glut1 and HK-II between WT MEFs and *Atg7*^−/−^ MEFs (left panel); quantitative analysis of protein expressions (right panel) cultured in high-glucose medium. *P<0.05, **P<0.01. (**c**) Protein expressions of Glut1 and HK-II in *Atg7*^−/−^ MEFs (left panel); quantitative analysis of protein expressions (right panel) treated with 0, 5, 10, 20 nM HF for 12 h in high-glucose medium. *P<0.05,**P<0.01. (**d**) Protein expressions of PCB and PCK2 in HCT116 and SW480 (upper panel); quantitative analysis of protein expressions (bottom panel) treated with 0, 5, 10, 20 nM HF for 2 h in EBSS medium. *P<0.05, **P<0.01. (**e**) Protein expressions of PCB and PCK2 between WT MEFs and *Atg7*^−/−^ MEFs (left panel); quantitative analysis of protein expressions (right panel) cultured in EBSS medium. *P<0.05, **P<0.01. (**f**) Protein expressions of PCB and PCK2 in *Atg7*^−/−^ MEFs (left panel); quantitative analysis of protein expressions (right panel) treated with 0, 5, 10, 20 nM HF for 2 h in EBSS medium. *P<0.05, **P<0.01

In our previous work, we found that HF can suppress glycolysis in high-glucose medium.^15^ Moreover, it should be noted that autophagy is affected by many factors including metabolic stress,^20, 21^ and HF dually regulates ATG7 for autophagosome formation. Therefore, we further asked if the autophagic-essential regulator ATG7 is required for the regulation of glucose metabolism in cells treated with HF under different nutrient conditions. To this end, we utilized *Atg7*^−/−^ MEFs for further interrogation to verify the pivotal role of autophagy in comparison with WT MEFs (Supplementary Figure S4). By performing cellular metabolome of MEFs, we identified lactate as the aerobic glycolysis marker in *Atg7*^−/−^ MEFs under nutrient-rich condition comparing with WT MEFs (Figure 5a). The elevated level of lactate in *Atg7*^−/−^ MEFs suggested that autophagy inhibits the Warburg effect under nutrient-rich condition. Glut1 and HK-II have been identified in our previous work, and HF downregulated the expressions of both targets.^15^ Here, we found that both Glut1 and HK-II were enhanced in *Atg7*^−/−^ MEFs to support our notion that *Atg7* is required for aerobic glycolysis modulation during nutrient-replete condition (Figure 5b). Intriguingly, HF treatment did not change the expressions of Glut1 and HK-II in *Atg7*^−/−^ MEFs, suggesting that ATG7 was essential for glycolytic regulation upon HF treatment (Figure 5c). Under nutrient-poor condition, however, gluconeogenesis would be an important route for cancer cell survival through autophagy.^22^ We further asked whether HF also could downregulate gluconeogenesis in CRC cells under nutrient-poor condition. By performing western blot assay of CRC cells cultured in EBSS medium, we found that HF downregulated pyruvate carboxylase (PCB) and mitochondrial phosphoenolpyruvate carboxykinase (PCK2), both of which are the key enzymes in gluconeogenesis (Figure 5d). The comparison of *Atg7*^−/−^ MEFs and WT MEFs also demonstrated that *Atg7* was essential for modulating gluconeogenesis (Figure 5e). The notion was further supported by HF treatment on *Atg7*^−/−^ MEFs, in which expressions of PCB and PCK2 did not change significantly (Figure 5f). These results indicate that HF regulates glycolysis/gluconeogenesis in an Atg7-dependent manner depending on nutritional conditions.

### HF dually regulates autophagy to retard tumor growth *in vivo*

**Figure 6:**
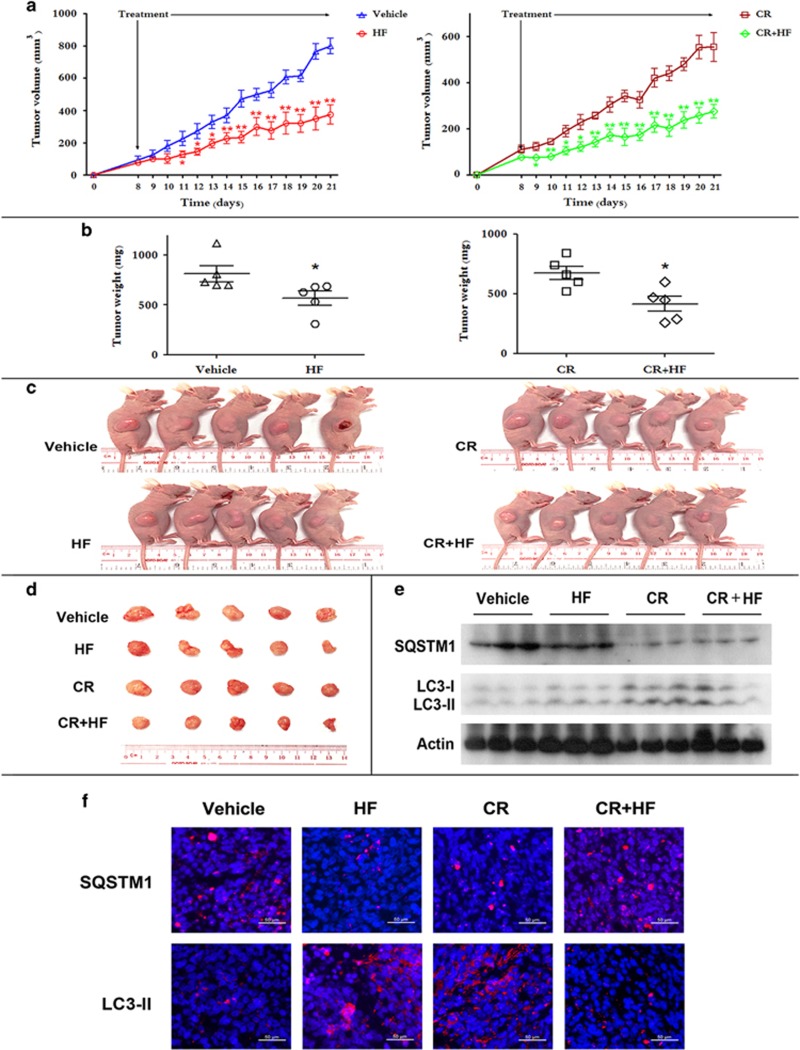
HF dually regulates autophagy for anti-CRC *in vivo*. (**a**) Six-week-old nude mice were engrafted with HCT116 cells and randomly divided into four groups: vehicle group, HF group, CR group, CR+HF group (*n*=5). Tumor volumes were calculated by the length and width measured by vernier calipers every day. *P<0.05, **P<0.01. (**b**) The tumor weights of the four groups. *P<0.05. (**c**) Photos of all the animals. (**d**)The xenograft tumors were dissected and measured. (**e**) Expression levels of SQSTM1 and LC3-II in xenograft tumors by western blot analysis. (**f**) IFC staining for SQSTM1 and LC3-II in xenograft tumors. Scale bar=50 *μ*m

As shown in cells cultured under both nutrient-rich and nutrient-poor conditions, HF dually regulates autophagy for anticancer *in vitro*. We further determined autophagic regulation for anticancer activity of HF *in vivo* by using nude mice inoculated with CRC cells. To conduct different conditions *in vivo*, caloric restriction (CR) was performed in mice fed with 70% of normal food intake. As a result, HF treatment retarded tumor growth in xenograft-bearing nude mice fed with standard chow diet (Figures 6a–d). It is well known that CR is one of the most important physiologic factors to induce autophagy.^23, 24^ Therefore, CR is mimicking poor nutrition *in vitro* with autophagic induction. We also found that HF retarded tumor growth in mice fed with 70% of normal food intake compared with CR control group (Figures 6a–d). Further assays by western blot and immunofluorescent imaging suggest that HF functions as an autophagic inducer in xenograft-bearing nude mice allowed access to food *ad libitum*, whereas an autophagic inhibitor in CR condition (Figures 6e and f). Together, HF treatment not only dually regulates autophagic flux in cancer cells *in vitro*, but also retards tumor growth *in vivo* through dual regulation of autophagy.

## Discussion

In conditions of metabolic stress, autophagy is activated, and cellular components are embedded into an autophagosome, providing an alternative way to maintain vital cellular activities.^25^ Autophagy is a double-edged sword in cancer, either impeding tumor initiation or promoting tumorigenesis.^26, 27, 28^ Although the detailed mechanism behind the role of autophagy in tumorigenesis still needs to be further investigated, here we provide straight forward evidence for understanding the dual role of HF in autophagy regulation in CRC depending on nutrient status. ULK1, a mammalian homolog of *Atg*1, modulates autophagy with different phosphorylation sites for its activities. The phosphorylation site at Ser757 is directly regulated by mTORC1 while phosphorylation sites at Ser317/Ser777 are directly regulated by AMPK.^17, 29^ In the initiation stage, intriguingly, HF induces autophagy through the Akt-mTORC1-ULK1 signaling pathway, whereas it inhibits autophagy via the LKB1-AMPK-ULK1 signaling pathway, indicating that HF not only impedes tumor initiation but also retards tumor growth through an autophagic pathway depending on nutritional status. To further interrogate autophagic flux, we used lysosomal inhibitor CQ, and performed confocal microscope of mRFP-GFP-LC3 puncta as well as MDC staining for a better visualization of elongation stage. At this stage, HF enhances *Atg7* expression under nutrient-rich condition but reduces *Atg7* expression under nutrient-poor condition to affect autophagosome formation. It is well known that *Atg7* is required for LC3 lipidation by conjugating to the lipid phosphatidylethanolamine and is subsequently recruited to both the outer and inner surfaces of the autophagosomal membrane.^14^ Thus, HF promotes autophagosome formation when nutrients are plentiful but blocks its formation under nutrient-poor condition. In the present study, HF was seen to facilitate autophagosome formation under nutrient-rich condition but impair lysosome function to prevent complete autophagic flux.

Of particular interest, by using *Atg7*^−/−^ MEFs, we found that elevated level of lactate and upregulation of Glut1 and HK-II in *Atg7*^−/−^ MEFs compared with WT MEFs, unveiling that autophagy deficiency either promotes glycolysis or limits intracellular recycling, which leads to compensational upregulation of glycolysis for energy homeostasis under nutrient-rich condition. In our previous work, we analyzed several glycolytic enzymes and found that Glut1 and HK-II are reduced with HF treatment in CRC cells cultured in high-glucose medium. However, the expressions of Glut1 and HK-II are not altered significantly with HF in *Atg7*^−/−^ MEFs, supporting the notion that autophagy is essential for glycolysis and HF inhibits glycolysis in an Atg7-dependent manner.

On the other hand, autophagy deficiency downregulates protein levels of PCB and PCK2 under nutrient-poor condition. Upon drug treatment, HF was also found to inhibit gluconeogenesis under nutrient-poor condition in an Atg7-dependent manner. Regarding both Glut1 and HK-II in glycolysis and both PCB and PCK2 in gluconeogenesis in autophagy-deficient MEFs upon HF treatment, it indicates that autophagy is essential for metabolic regulation in mammalian cells upon HF treatment. The obtained results in present study suggest a new candidate for CRC treatment although the metabolic reprogramming by autophagy upon HF treatment still needs to be further explored. It has been reported CR affects several physiologic pathways including gluconeogenesis in multiple tissues,^30^ even enhances anticancer immunosurveillance.^31^ Nonetheless, HF still can retard tumor growth in xenograft-bearing nude mice with CR condition, supporting our notion that HF exerts dual role in modulating autophagy for retarding tumor growth *in vivo*.

**Figure 7:**
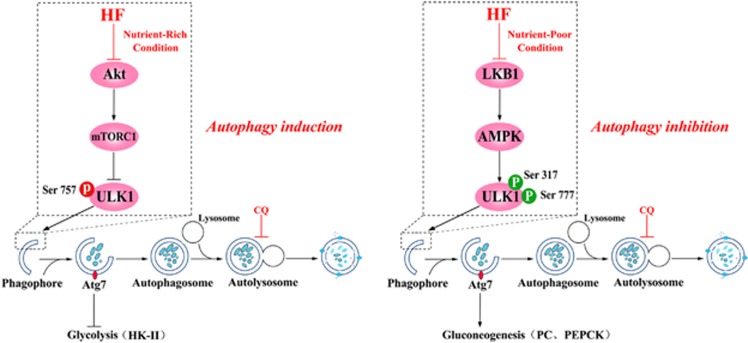
Proposed mechanism of HF targeting autophagy and metabolism in CRC cells. HF dually regulates autophagy and glucose metabolism for its anticancer activity through Akt-mTORC1-ULK1 or LKB1-AMPK-ULK1 signaling pathway. More importantly, HF impairs glycolysis or gluconeogenesis via an Atg7-dependent manner, indicating that autophagy is essential for metabolic regulation of HF treatment

HF induces or inhibits autophagy through modulation of Akt-mTORC1-ULK1 or LKB1-AMPK-ULK1 signaling pathway presented at the current work in combination with our previous report.^15^ Our investigations of autophagic flux also validated the hypothesis that HF can dually regulate elongation stage for autophagosome formation. Moreover, glycolysis or gluconeogenesis is inhibited in cancer cells via the autophagic pathway upon HF treatment, providing a promising approach for anticancer by targeting autophagic pathway. Therefore, our proposed working model is represented in Figure 7. *Atg7* has a critical role in metabolic regulation in mammalian cells upon HF treatment, although other autophagy-related genes are still need to be investigated in the future.

## Materials and methods

### Chemicals and reagents

HF hydrobromide, MDC, EBSS, CQ, Pierce (R) BCA Protein Assay Kit and SBI-0206965 were obtained from Sigma-Aldrich (Munich, Germany). Antibodies against SQSTM1/p62, LC3-II, phosphor-ULK1 (Ser317), phosphor-ULK1 (Ser757), phosphor-mTOR (Ser2448), Glut1, hexokinase II, PCK2, PCB, phosphor-AMPK*α*, AMPK*α*, phosphor-ACC*α* (Ser79), ACC*α*, ATG7, phosphor-LKB1, LKB1, phosphor-CaMKK*β* (Ser511), and *β*-actin were purchased from Cell Signaling Technology, Inc. (Danvers, MA, USA). Antibodies against phosphor-ULK1 (Ser777), CaMKK*β,* and goat anti-mouse IgG-HRP secondary antibody were purchased from Santa Cruz Biotechnology (Santa Cruz, CA, USA). HRP-goat anti-rabbit secondary antibody was purchased from Invitrogen (Carlsbad, CA, USA).

### Cell culture

HCT116 and SW480 were purchased from American Type Culture Collection (Manassas, VA, USA). WT MEFs and Atg7^-/-^ MEFs were kindly provided by Professor Kevin Ryan in Beatson Institute for Cancer Research. All cells were cultured in DMEM (Gibco/Invitrogen, 12800-017) supplemented with 10% FBS (PAA, A15-101), 10 U/ml penicillin–streptomycin (Gibco/Invitrogen, 15140-122) in a humidified atmosphere containing 10% CO_2_ and 90% air at 37 °C. The medium was changed every 3 days, and cells were passaged using 0.05% trypsin/EDTA. For nutrient starvation, cells cultured in DMEM were washed three times with PBS, and then cultured in EBSS medium for 2 h.

### GFP-LC3-II and mRFP-GFP-LC3 translocation and imaging in living cells

HCT116 cells were transfected with pEGFP-LC3-II and mRFP-GFP-LC3 plasmids using lipofectamine 2000, respectively (Invitrogen, 11668-019). One day after transfection, cells were treated with 20 nM HF for 12 h in high-glucose medium and for 2 h in EBSS medium prior to fixation, respectively. Then cells were imaged for GFP and RFP by using a Leica TCS SP8 (Leica) confocal microscope in different channels (GFP: excitation wavelength 488 nm, emission filter 500~550 nm; RFP: excitation wavelength 552 nm, emission filter 580~620 nm).

### Western blot analysis

After treated with 20 nM HF in high-glucose medium for 12 h and in EBSS medium for 2 h, respectively, cells were suspended in lysis buffer to obtain whole cell lysates. Following centrifugation at 13 500 × *g* for 15 min at 4 °C, total protein concentration was measured using a Pierce(R) BCA Protein Assay Kit. 10–25 *μ*g of protein was separated on 10% sodium dodecylsulphate-polyacrylamide gel and transferred onto polyvinylidene difluoride membranes. After blocking (5% skim milk powder in TBS-Tween 20) for 1 h at room temperature, the membranes were then incubated with primary antibody overnight at 4 °C. The membranes were incubated with secondary antibody for 1 h at room temperature. All antibodies were diluted in TBS-Tween 20 containing 5% dry milk. The immune-reactive proteins were detected by enhanced chemiluminescence (ECL) using X-ray film and ECL reagent.

### *In vivo* xenograft studies

Male BALB/c nude mice, 6-weeks old, were obtained from the Laboratory Animal Services Centre, The Chinese University of Hong Kong. Mice were kept at room temperature 23± 2 °C with an alternating 12-h light–dark cycle, and were allowed access to food and water *ad libitum*. All of the experimental protocols were carried out with the approval of the Committee on Use of Human and Animal Subjects in Teaching and Research of Hong Kong Baptist University and according to the Regulations of the Department of Health, Hong Kong SAR, China. HCT116 cells (8 × 10^6^ cells per mouse) were suspended in PBS and inoculated subcutaneously into the right flank of each mouse, and tumor growth was monitored regularly. Once tumors were palpable (~100 mm^3^), mice were divided at random into four groups with five mice in each group. The groups were as follows: (1) vehicle group in nutrient-rich condition, normally fed, receiving daily i.p. saline; (2) HF group in nutrient-rich condition, normally fed, receiving daily i.p. 0.1 mg/kg of HF; (3) vehicle group in nutrient-poor condition, fed with 70% of their normal food intake, receiving daily i.p. saline; (4) HF group in nutrient-poor condition, fed with 70% of their normal food intake, receiving daily i.p. 0.1 mg/kg of HF. The tumors were measured with calipers every day, and the tumor volumes were calculated by the following formula: *a*^2^ × *b* × 0.4, where ‘*a*’ is the smallest diameter and ‘*b*’ is the diameter perpendicular to ‘*a*’. Other indicators of general health, such as body weight, feeding behavior, and motor activity of each animal, were also monitored. After administration of HF or saline for 2 weeks, the mice were euthanized, and the tumor xenografts were immediately dissected, weighed, stored, and fixed.

### Protein extraction from tumors for western blot analysis

To measure the SQSTM1/p62 and LC3-II activity in tumor tissue, three tumors in every group were collected and dispersed in lysis buffer by sonication for protein extraction. After centrifuging at 13 500 × *g* for 15 min at 4 °C, the supernatants were collected and regarded as the total soluble proteins subsequently used for western blot analysis.

### Immunofluorescence assay

Xenograft tumors were resected immediately and fixed in 10% neutral buffered paraformaldehyde at 4 °C for 24 h. Selected samples were embedded in paraffin, sectioned and stained with SQSTM1/p62 and LC3-II. All primary antibodies were used for dilution at 1:100. After overnight incubation at 4 °C, the sections were incubated with flurochrome-conjugated secondary antibody for 1 h and stained with DAPI for 10 min. The sections were then mounted with DPX mountant (Sigma-Aldrich, 317616) for analysis.

### Statistical analysis

Each experiment was performed at least three times. GraphPad Prism 5.0 software was used for statistical analysis. The values of *P*<0.05 were considered as statistically significant.
