# Supplementary material for: Halofuginone dually regulates autophagic flux through nutrient-sensing pathways in colorectal cancer
Source: Cell Death Dis. 2017 May 11;8(5):e2789–. doi: 10.1038/cddis.2017.203 (PMC5520722; doi:10.1038/cddis.2017.203)
Supplement: Supplementary Information [file cddis2017203x1.docx]

Supplementary information

**Halofuginone Dually Regulates Autophagic Flux through Nutrient-Sensing Pathways in Colorectal Cancer**

Guo-Qing Chen^1,2^, Rui-Hong Gong^1^, Da-Jian Yang^2^, Ge Zhang^1^, Ai-Ping Lu^1^, Siu-Cheong Yan^3^, Shu-Hai Lin^1,4,^*, Zhao-Xiang Bian^1,^*

^1^ Laboratory of Brain and Gut Research, Center for Clinical Research on Chinese Medicine, School of Chinese Medicine, Hong Kong Baptist University, Hong Kong SAR, China.

^2^ Chongqing Academy of Chinese Materia Medica, Chongqing, China.

^3^ Department of Applied Biology and Chemical Technology, Hong Kong Polytechnic University, Hung Hom, Kowloon, Hong Kong.

^4^ Department of Biochemistry and Molecular Cell Biology, Shanghai Key Laboratory for Tumor Microenvironment and Inflammation, Shanghai Jiao Tong University School of Medicine (SJTU-SM), Shanghai 200025, China.

* To whom correspondence should be addressed: [bzxiang@hkbu.edu.hk](mailto:bzxiang@hkbu.edu.hk) (Z.X.B.) or [slin@shsmu.edu.cn](mailto:slin@shsmu.edu.cn) (S.H.L.).

**Material and Methods**

### Staining of autophagic vacuoles by MDC

Fluorescent probe MDC is a selective marker for acidic vesicular organelles used to evaluate autophagy. HCT116 cells were cultured with 20 nM HF in high glucose medium for 12 h and EBSS medium for 2 h, respectively, the cells were then incubated with 0.05 mM MDC in PBS at 37 °C for 10 min. After incubation, cells were washed four times with PBS and immediately viewed on a Leica TCS SP8 (Leica) confocal microscope (excitation wavelength 405 nm, emission filter 500～560 nm).

### Quantitative analysis of mRNA

Total RNA was isolated from cells using TRIzol reagent (Invitrogen). The cDNAs were prepared by reverse transcription. Quantitative polymerase chain reaction (PCR) was performed using the QuantiTect SYBR green PCR Master mix (Qiagen, Valencia, CA) with 1µL cDNA in a final volume of 10 µL and the following primers at final concentrations of 1000 nM. Primers for *Atg7* were5’-TGTGCCTCACCAGGTTCTTGAT-3’ (forward) and 5’-GGTGGAAATCTGGCGTCACT-3’ (reverse). Primers for *Atg5* were 5’-CAAGGTGGAGTTGGCGAGA-3’ (forward) and 5’-CCAAAGCCAAACTTAGTAAGCA-3’ (reverse). Primers for *Atg10* were5’-CCATGGAGGAGGCTTTCGAG-3’ (forward) and 5’-AGTGTCCCATGGTCCCTGTA-3’ (reverse). Primers for *Atg12* were5’-TGCTGGAGGGGAAGGACTTA-3’ (forward) and 5’-CCATCACTGCCAAAACACTCA-3’ (reverse). Amplification of cDNAs was performed using a Light Cycler 2000 (Roche, Indianapolis, IN). The cycling program comprised a denaturation step for 15 minutes at 95 °C, followed by 40 cycles of denaturation (95 °C for 15 seconds), annealing (59 °C for 20 seconds), and extension (72 °C for 15 seconds). After amplification, a melting curve analysis was performed with denaturation at 95 °C for 5seconds, then continuous fluorescence measurement from 70 °C to 95 °C at 0.1 °C/second. Each sample was amplified in duplicate.

### Cell culture and metabolite extraction

Cells was seeded into 10 cm dishes at a density of 5×10^6^ cells per dish in 5 mL medium, which comprised high-glucose DMEM supplemented with 10% FBS and 10 U/ml penicillin-streptomycin. After culturing for 24 h, the nutrient-rich high glucose DMEM was removed, and cells were briefly rinsed with PBS twice. Then cells were cultured in high glucose DMEM containing 20 nM HF for 12 h, which compared to control group without HF treatment. After treatment, culture medium was removed and cells were rapidly rinsed with PBS twice. The residual PBS was removed by vacuum suction. Cells were then quenched using 1.5 mL cold HPLC-grade methanol. Next, cells were quickly detached from the culture dish using a cell lifter. The methanol solution containing the quenched cells was pipetted into a 2-mL centrifuge tube. Samples were then vortexed-mixed for 30 sec and submerged for 1 min in liquid nitrogen. Samples were then thawed in ice. This process was repeated three times and samples were centrifuged at 10,000 g for 10 min at 4 °C. The supernatant was removed into a new tube, and cold 0.5 mL 80% methanol was added to the residue for vortexed-mixing for 30 sec; samples were then centrifuged at 10,000 g for 10 min at 4 °C. Finally, the two extraction solutions were pooled in a tube for evaporation till dryness under airflow and stored at ‒80 °C till analysis.

### NMR analysis

Samples were analyzed on a Bruker AVIII 600 MHz spectrometer operating at 600.13 MHz equipped with a double resonance 5-mm BBFO probe at 298 K. One dimensional ^1^H Carr–Purcell–Meiboom–Gill (CPMG) NMR experimental results were acquired using a Bruker standard CPMG pulse sequencer (cpmgpr1d). 1ms individual CPMG spin echo was repeated 20 times, resulting in a total spin-spin relaxation delay of 20 ms. The CPMG sequence-enabled spectra edited by T_2_ relaxation times removed the broad signal associated with high molecular weight macromolecules, facilitating the observation of low molecular weight metabolites. 64 to 256 free induction decays (FIDs) were collected into 16 K data points with a spectral width of 9615.38 Hz (16 ppm). A 4-s relaxation delay was used between pulses. Suppression of water was achieved by weak irradiation during the relaxation delay. Spectra were analyzed in Topspin (Bruker Biospin Corporation). FIDs were zero-filled to 32K and an exponential line-broadening function of 0.3 Hz was applied to the FID prior to Fourier transformation. All sample spectra were phased and manually baseline-corrected, and spectra were referenced to the methyl doublet of lactate at 1.33 ppm.

**Supplementary Figures**

Figure S1.


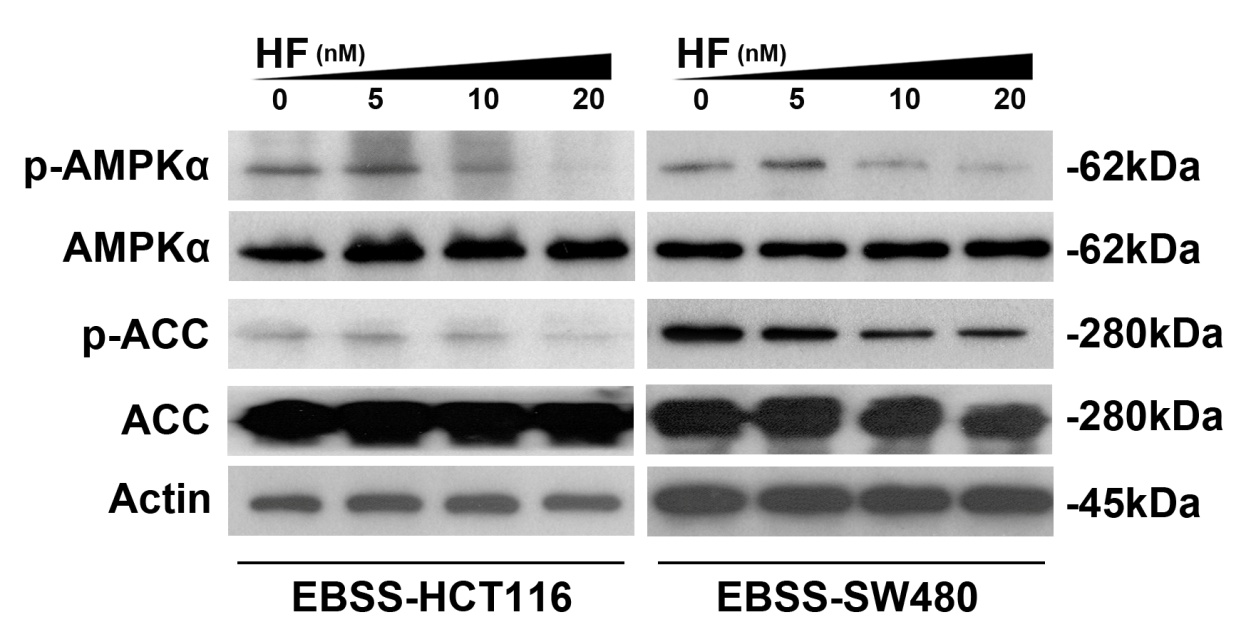


Figure S1. Protein expressions of phospho-AMPK and phospho-ACC in HCT116 and SW480 cells treated with 0, 5, 10, 20 nM HF for 2 h in EBSS medium.

Figure S2.


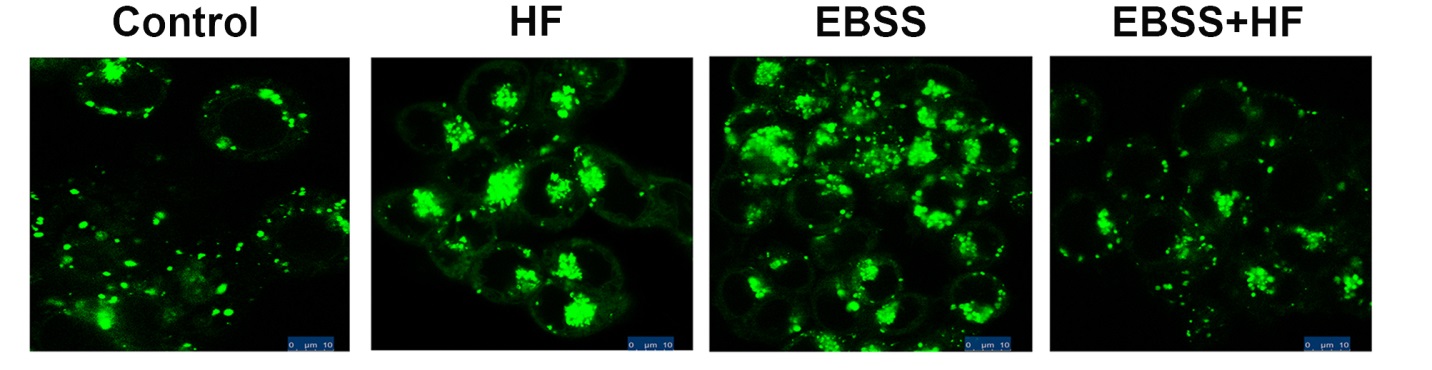


Figure S2. HCT116 cells were treated with 20 nM HF separately for 12 h in high glucose medium or 2 h in EBSS medium, then cells were staining with MDC and observed by confocal microscope. Scale bar: 10 μm.

Figure S3.


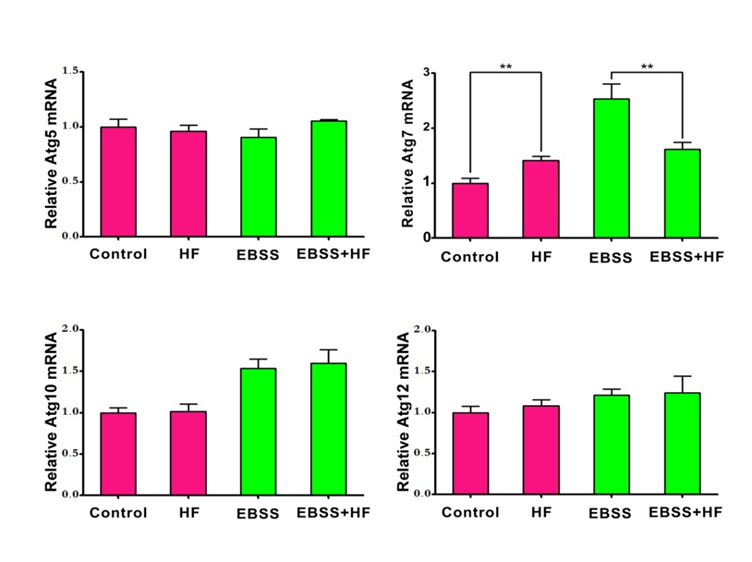


Figure S3. HCT116 cells were treated with 20 nM HF separately for 12 h in high glucose medium or 2 h in EBSS medium, and then mRNA levels of *Atg5*, *Atg7*, *Atg10* and *Atg12* in cells were analyzed by RT-PCR. *P < 0.05, **P < 0.01.

Figure S4.


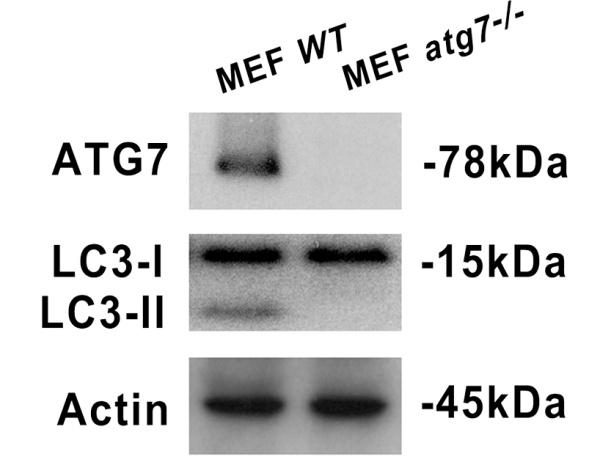


Figure S4. *Atg7*^-/-^ MEFs and WT MEFs were analyzed by western blot for ATG7 and LC3-II.
